# Supplementary material for: Dysbiosis-associated gut bacterium Ruminococcus gnavus varies at the strain level in its ability to utilize key mucin component sialic acid
Source: Microbiol Spectr. 2025 Jul 11;13(8):e03090-24. doi: 10.1128/spectrum.03090-24 (PMC12323340; doi:10.1128/spectrum.03090-24)
Supplement: Supplemental materials — Fig. S1 to S6, Tables S1 to S4, supplemental materials and methods. [file spectrum.03090-24-s0001.docx]

**SUPPLEMENTARY INFORMATION**

Dysbiosis-associated gut bacterium *Ruminococcus gnavus* varies at the strain level in ability to utilize key mucin component sialic acid

Olga M. Sokolovskaya^1^*, Jasmina Uzunovic^2†^, Yutian Peng^1^, Mikiko Okumura^3^, Lingjue Mike Wang^4^, Yuhui Zhou^3^, Zijuan Lai^4^, Elizabeth Skippington^2^, Man-Wah Tan^1^

^1^Department of Infectious Diseases & Host-Microbe Interactions, Genentech, Inc., South San Francisco, CA, United States

^2^Department of OMNI Bioinformatics, Genentech, Inc., South San Francisco, CA, United States

^3^Department of Discovery Chemistry, Genentech, Inc., South San Francisco, CA, United States

^4^Department of Drug Metabolism and Pharmacokinetics, Genentech, Inc., South San Francisco, CA, United States

^†^Present affiliation: Department of DSS Hematology, Roche, Canada

*Correspondence to olga.sokolovskaya.phd@gmail.com

**Supplementary Figures**

**
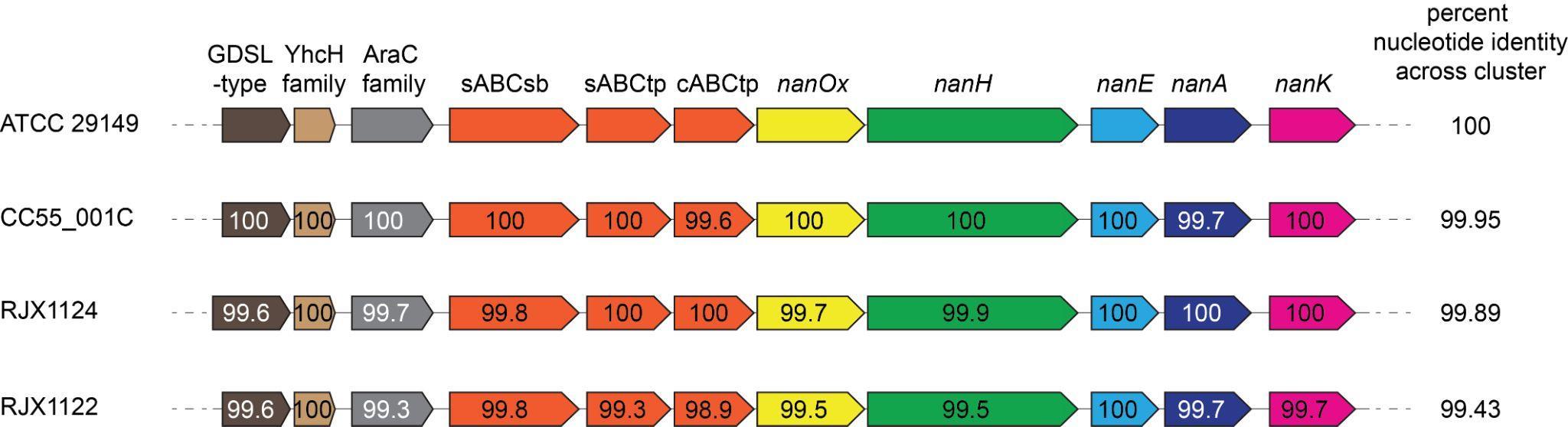
**

Supplementary Figure 1. Synteny and sequence identity of the *nan* cluster across diverse *R. gnavus* strains. Four strains sampling the phylogenetic diversity of the *R. gnavus* clade were selected for this analysis. The overall nucleotide identity of this cluster relative to ATCC 29149 is recorded on the right. The numbers inside of each open reading frame indicate the percent identity of each encoded protein at the amino acid level, relative to ATCC 29149.

**
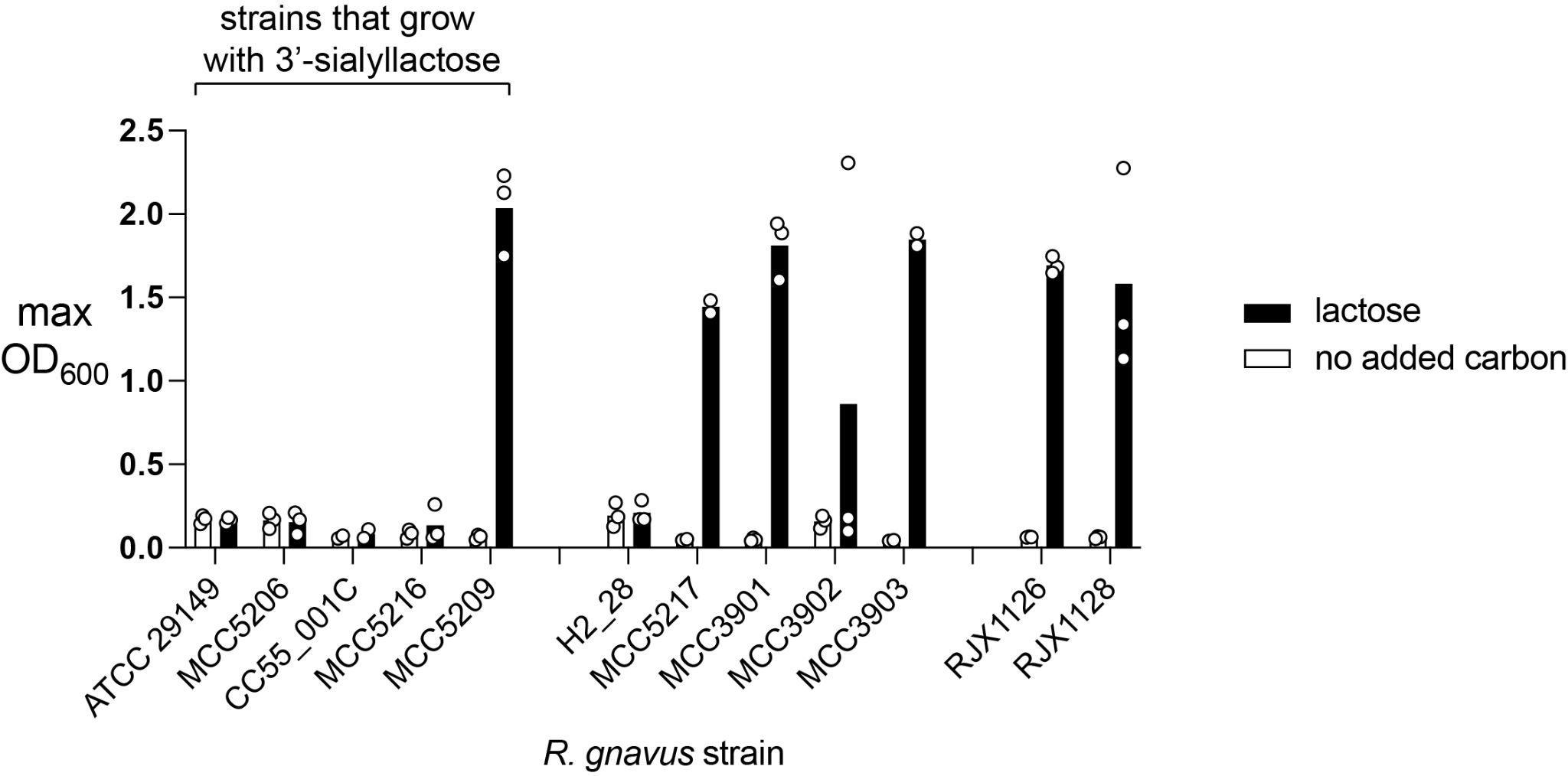
**

Supplementary Figure 2. *R. gnavus* strain growth in basal YCFA medium supplemented with lactose. Bars indicate the average of at least two independent experiments, each represented by a single data point.


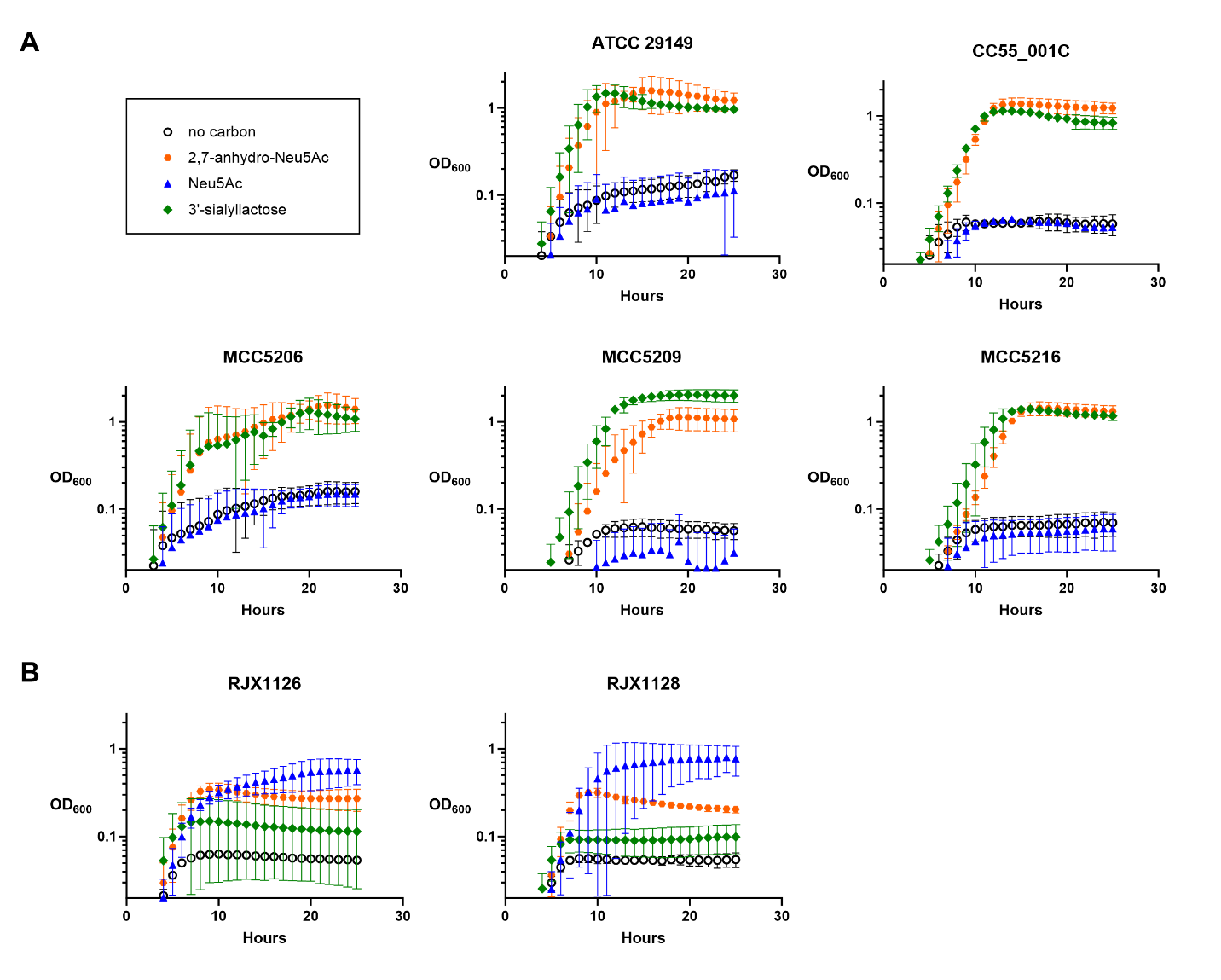


Supplementary Figure 3. *R. gnavus* strain growth in basal YCFA medium supplemented with simple sugars. (A) 2,7-anhydro-Neu5Ac metabolizing strains, (B) Neu5Ac catabolizing strains. Data points and error bars indicate the average and standard deviation, respectively, of at least two independent experiments


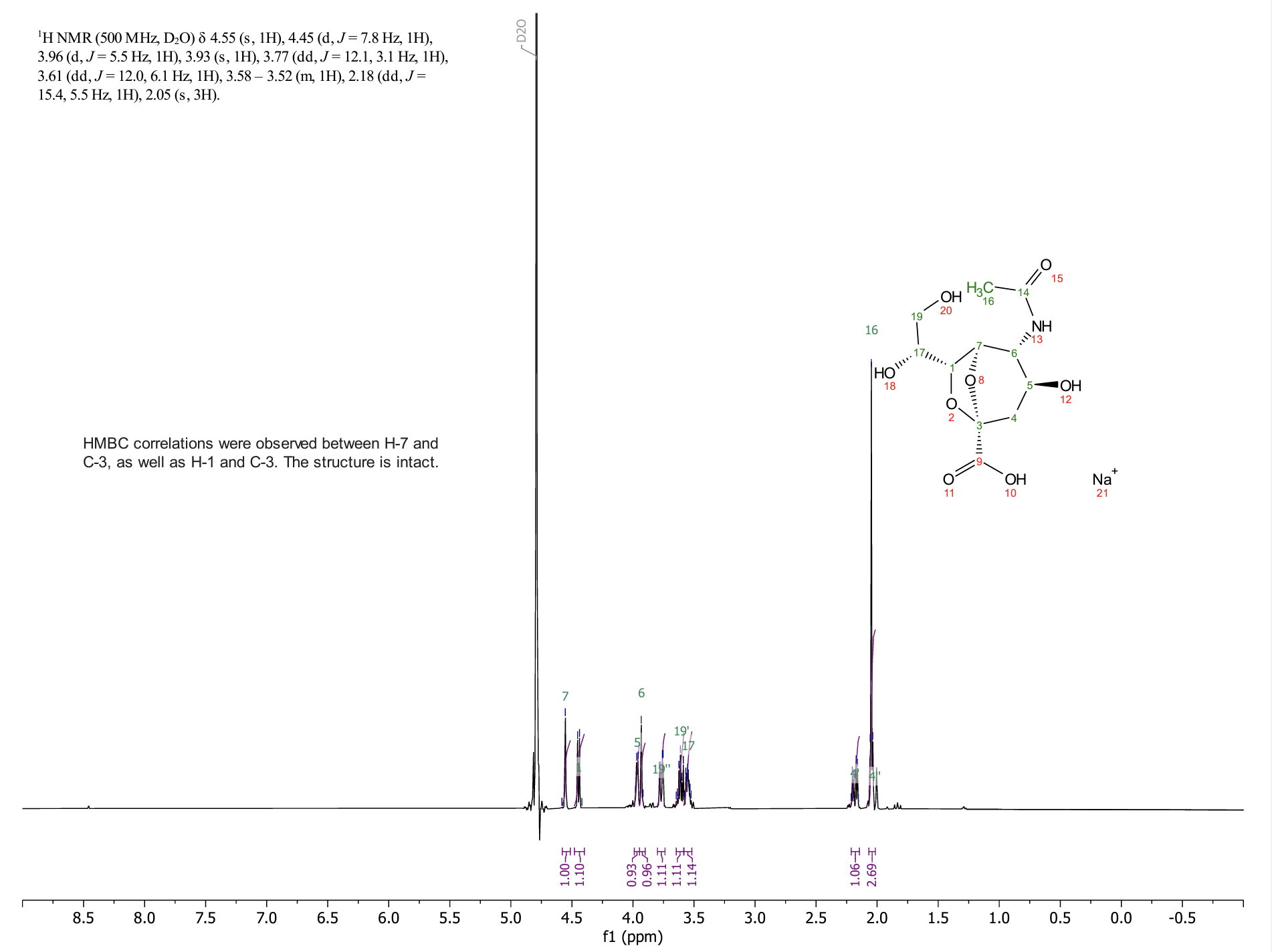


**A**


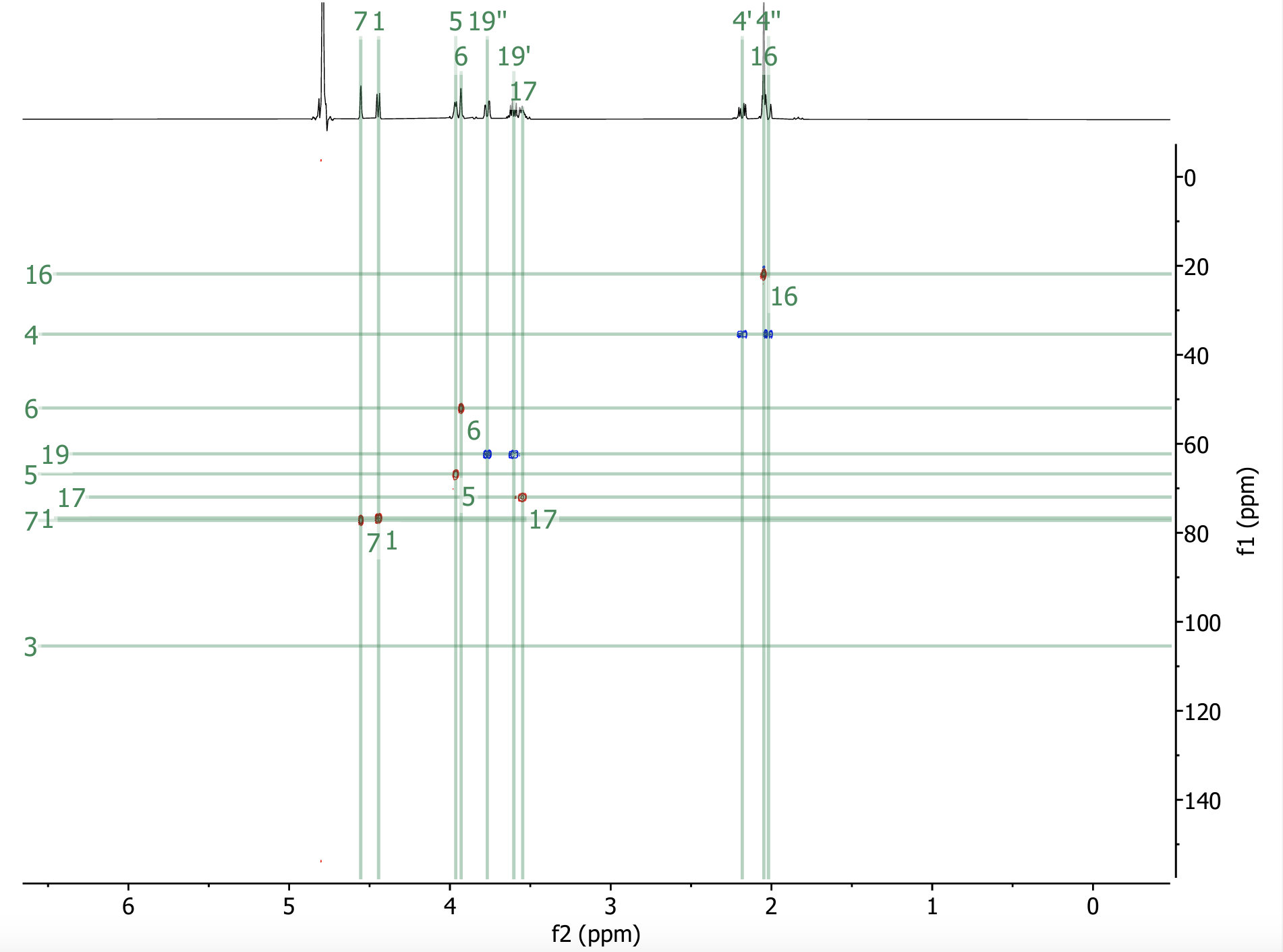


**B**


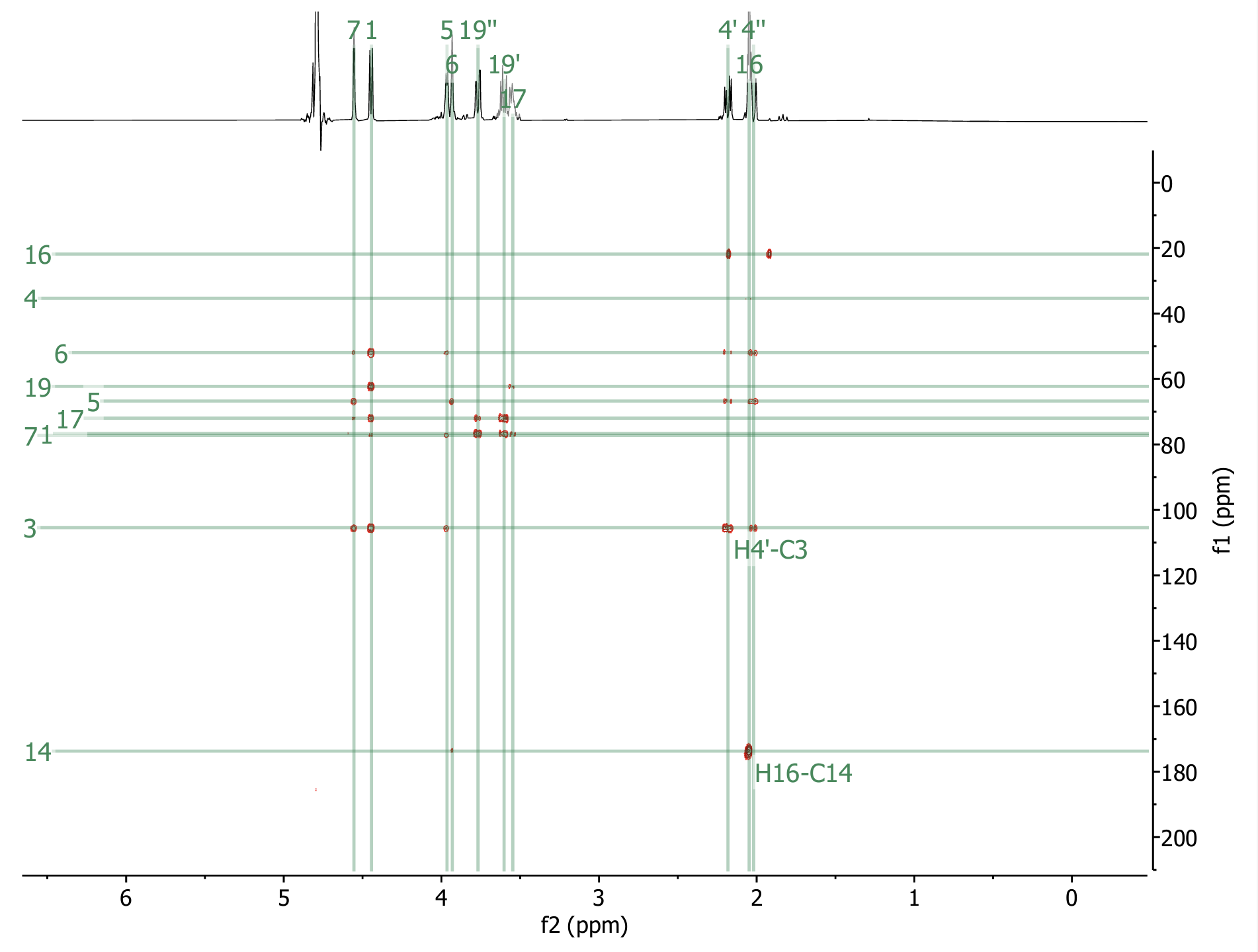

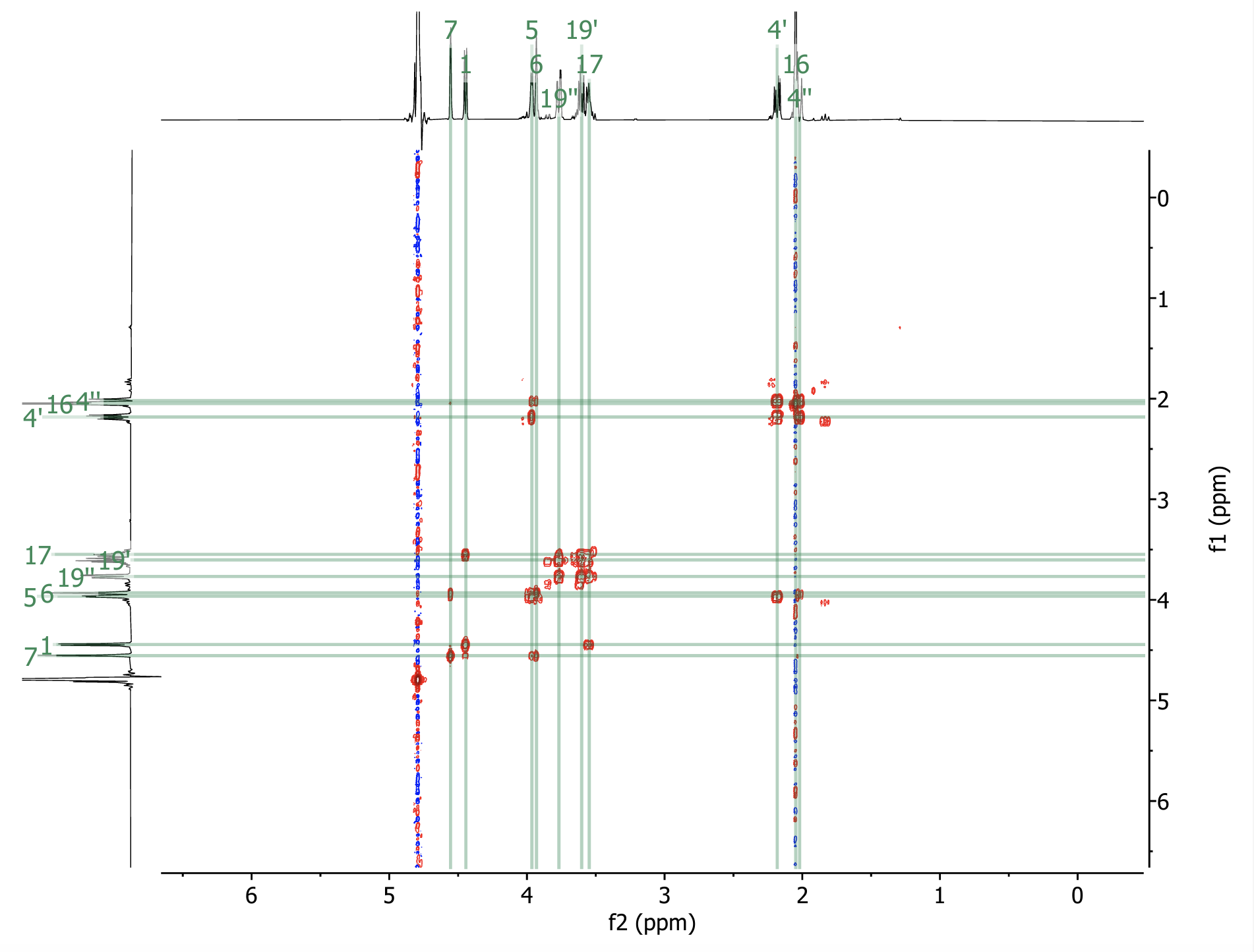


**C**

**D**

**C**


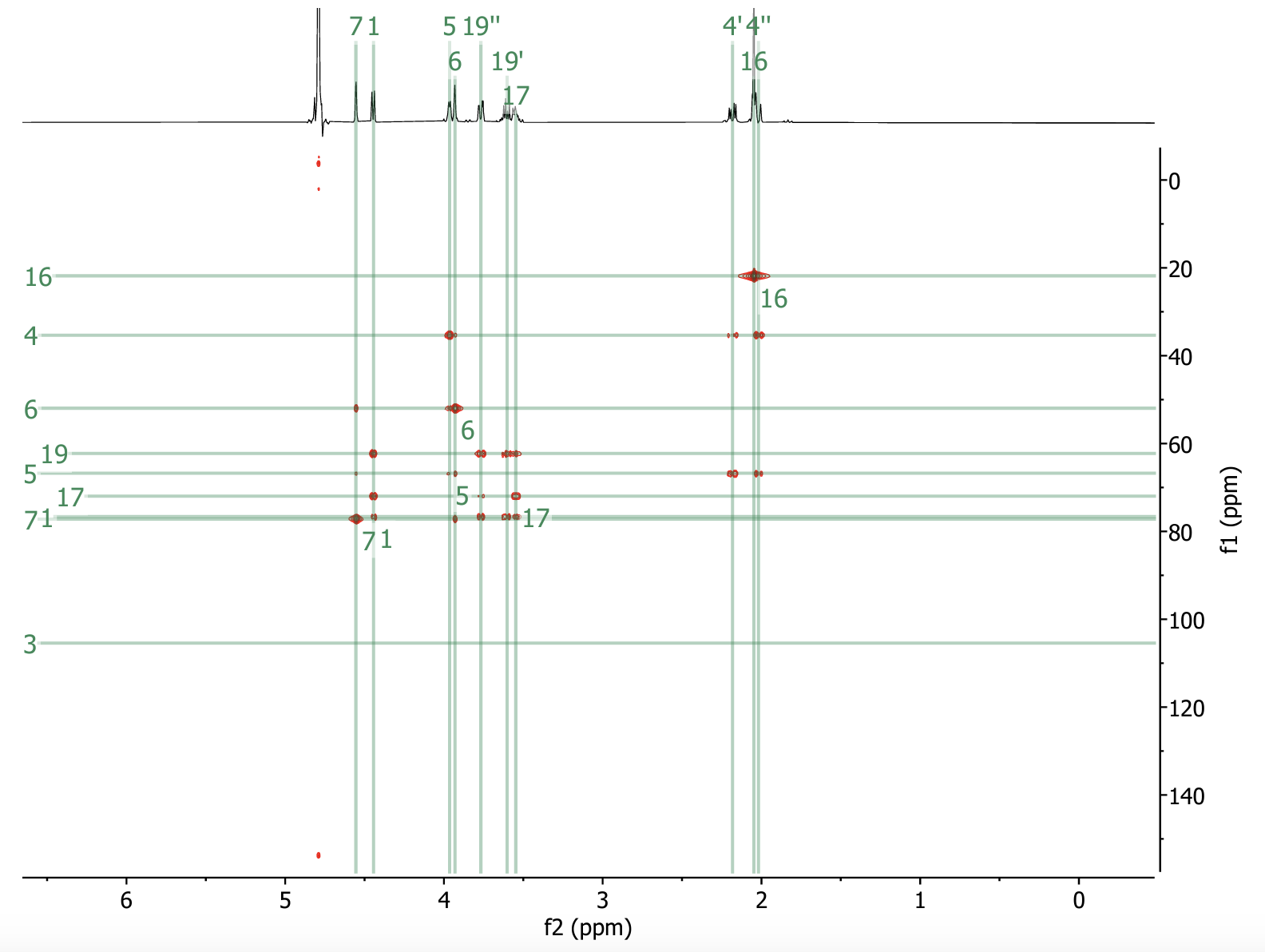


**E**

Supplementary Figure 4. (A-E) Two-dimensional NMR analysis of 2,7-anhydro-Neu5Ac synthesized in this study.


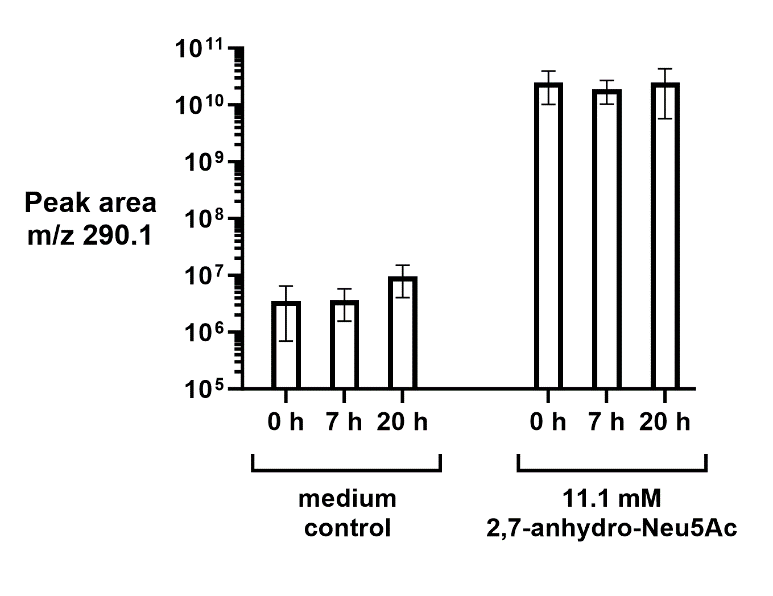


Supplementary Figure 5. Stability of 2,7-anhydro-Neu5Ac in bYCFA medium at 37 degrees, as demonstrated by liquid chromatography-mass spectrometry (LC-MS)-based detection of 2,7-anhydro-Neu5Ac in the medium at various time points. Bars and error brackets represent the mean and standard deviation, respectively, of three technical replicates. For method details, see Supplementary Materials and Methods.


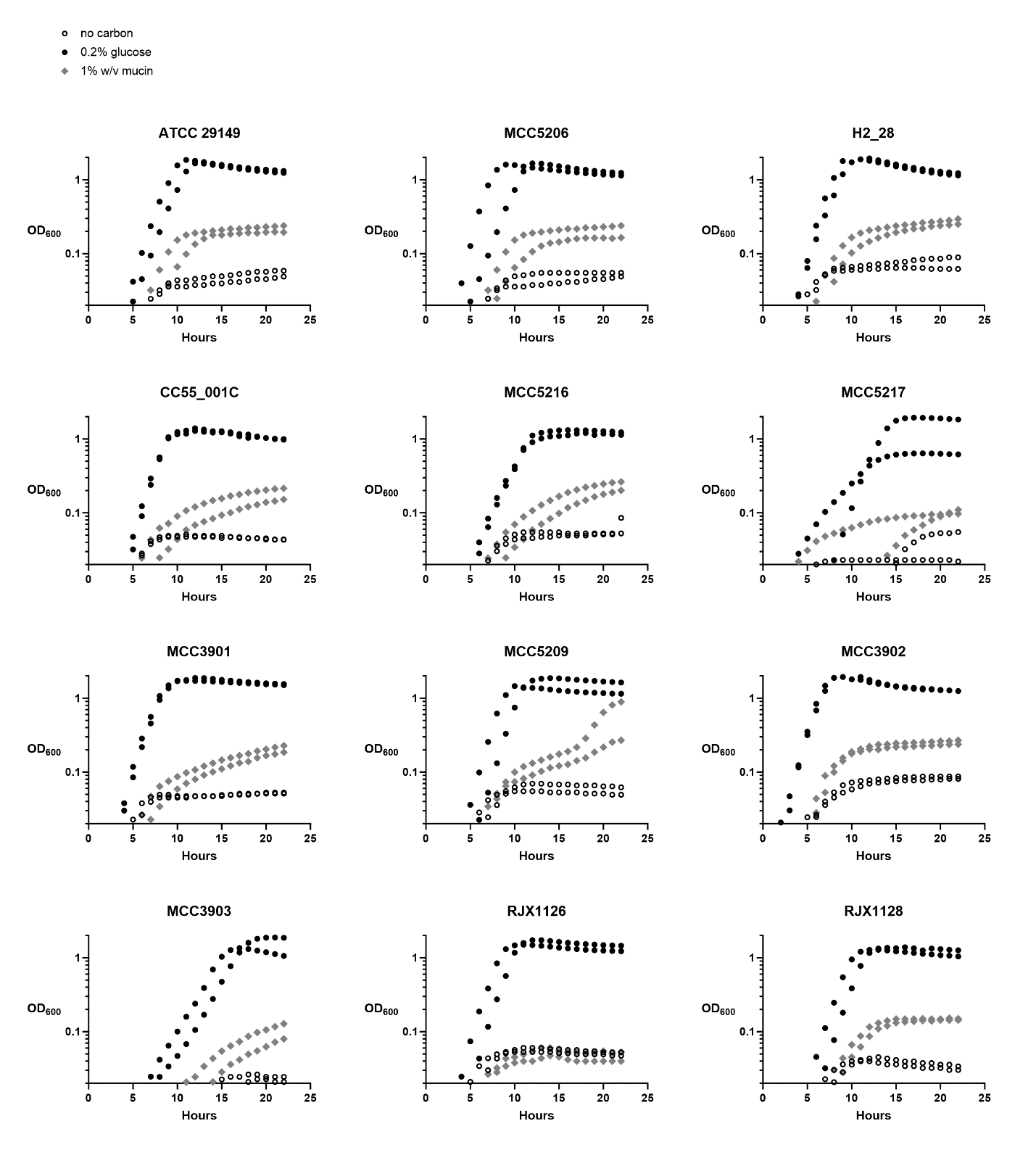


Supplementary Figure 6. *R. gnavus* strain growth in basal YCFA medium supplemented with purified porcine gastric mucin (pPGM). Data points represent two independent experiments.

**Supplementary Tables**

Supplementary Table 1. Bacterial *s*trains cultured in this study.

| Strain name | Species | Source |
| --- | --- | --- |
| ATCC 29149 | *Ruminococcus gnavus* | American Type Culture Collection (ATCC)^1^ |
| CC55_001C (HM-1056) | *Ruminococcus gnavus* | BEI Resources^2^ |
| H2_28 (DSM108212) | *Ruminococcus gnavus* | Leibniz Institute DSMZ - German Collection of Microorganisms and Cell Cultures GmbH |
| MCC3901 | *Ruminococcus gnavus* | Etrolizumab clinical trial; UC patient; stool^3,4^ |
| MCC3902 | *Ruminococcus gnavus* | Etrolizumab clinical trial; UC patient; stool^3,4^ |
| MCC3903 | *Ruminococcus gnavus* | Etrolizumab clinical trial; CD patient; stool^3,4^ |
| MCC5206 | *Ruminococcus gnavus* | Etrolizumab clinical trial; CD patient; stool^3,4^ |
| MCC5209 | *Ruminococcus gnavus* | Etrolizumab clinical trial; CD patient; stool^3,4^ |
| MCC5216 | *Ruminococcus gnavus* | Etrolizumab clinical trial; UC patient; stool^3,4^ |
| MCC5217 | *Ruminococcus gnavus* | Etrolizumab clinical trial; CD patient; stool^3,4^ |
| RJX1126 | *Ruminococcus gnavus* | Laboratory of Ramnik Xavier^5^ |
| RJX1128 | *Ruminococcus gnavus* | Laboratory of Ramnik Xavier^5^ |

Supplementary Table 2. *Ruminococcus (Mediterraneibacter) gnavus* genomes included in phylogenetic tree construction.

| GenBank Accession | Assembly Name | Strain name | Assembly Level | Assembly Release Date | WGS project accession | No. of Scaffolds |
| --- | --- | --- | --- | --- | --- | --- |
| GCA_002865285.1 | ASM286528v1 | RJX1128 | Scaffold | 1/5/2018 | NIHW01 | 97 |
| GCA_002865305.1 | ASM286530v1 | RJX1126 | Scaffold | 1/5/2018 | NIHU01 | 58 |
| GCA_002865325.1 | ASM286532v1 | RJX1124 | Scaffold | 1/5/2018 | NIHS01 | 126 |
| GCA_002865345.1 | ASM286534v1 | RJX1125 | Scaffold | 1/5/2018 | NIHT01 | 92 |
| GCA_002865355.1 | ASM286535v1 | RJX1123 | Scaffold | 1/5/2018 | NIHR01 | 120 |
| GCA_002865385.1 | ASM286538v1 | RJX1127 | Scaffold | 1/5/2018 | NIHV01 | 59 |
| GCA_002865405.1 | ASM286540v1 | RJX1120 | Scaffold | 1/5/2018 | NIHO01 | 132 |
| GCA_002865425.1 | ASM286542v1 | RJX1122 | Scaffold | 1/5/2018 | NIHQ01 | 87 |
| GCA_002865435.1 | ASM286543v1 | RJX1119 | Scaffold | 1/5/2018 | NIHN01 | 72 |
| GCA_002865465.1 | ASM286546v1 | RJX1121 | Scaffold | 1/5/2018 | NIHP01 | 105 |
| GCA_002865485.1 | ASM286548v1 | RJX1118 | Scaffold | 1/5/2018 | NIHM01 | 78 |
| GCA_003436535.1 | ASM343653v1 | TM04-16 | Scaffold | 8/31/2018 | QSPZ01 | 90 |
| GCA_003438585.1 | ASM343858v1 | TF01-20-2 | Scaffold | 8/31/2018 | QSSX01 | 234 |
| GCA_003457855.1 | ASM345785v1 | AF27-4BH | Scaffold | 9/5/2018 | QRTJ01 | 144 |
| GCA_003459125.1 | ASM345912v1 | AF24-23LB | Scaffold | 9/5/2018 | QRUR01 | 44 |
| GCA_003459705.1 | ASM345970v1 | AF19-16AC | Scaffold | 9/5/2018 | QRWQ01 | 72 |
| GCA_003464115.1 | ASM346411v1 | AM51-4BH | Scaffold | 9/6/2018 | QSDZ01 | 139 |
| GCA_003464875.1 | ASM346487v1 | AF13-14A | Scaffold | 9/6/2018 | QSAA01 | 132 |
| GCA_003467625.1 | ASM346762v1 | AM38-12 | Scaffold | 9/6/2018 | QSHH01 | 52 |
| GCA_003468635.1 | ASM346863v1 | AM32-6 | Scaffold | 9/6/2018 | QSIR01 | 108 |
| GCA_003469585.1 | ASM346958v1 | AM27-32 | Scaffold | 9/6/2018 | QSKP01 | 66 |
| GCA_003470265.1 | ASM347026v1 | AM25-19 | Scaffold | 9/6/2018 | QRHH01 | 63 |
| GCA_003470485.1 | ASM347048v1 | AM21-41 | Scaffold | 9/6/2018 | QRIP01 | 154 |
| GCA_003471005.1 | ASM347100v1 | AM22-7AC | Scaffold | 9/6/2018 | QRIA01 | 62 |
| GCA_003471265.1 | ASM347126v1 | AM21-18 | Scaffold | 9/6/2018 | QRIS01 | 151 |
| GCA_003471625.1 | ASM347162v1 | AM17-51 | Scaffold | 9/6/2018 | QRJQ01 | 67 |
| GCA_003473045.1 | ASM347304v1 | AM13-39 | Scaffold | 9/6/2018 | QRLD01 | 58 |
| GCA_003473185.1 | ASM347318v1 | AM12-54 | Scaffold | 9/6/2018 | QRLN01 | 52 |
| GCA_003475065.1 | ASM347506v1 | AF34-4BH | Scaffold | 9/6/2018 | QRPU01 | 68 |
| GCA_003475365.1 | ASM347536v1 | AF33-12 | Scaffold | 9/6/2018 | QRQE01 | 197 |
| GCA_008121495.1 | ASM812149v1 | JCM6515 | Complete Genome | 8/28/2019 |  | 1 |
| GCA_009831375.1 | ASM983137v1 | ATCC 29149 | Complete Genome | 1/6/2020 |  | 1 |
| GCA_013299595.1 | ASM1329959v1 | MSK.5.19 | Contig | 6/8/2020 | JAAILN01 |  |
| GCA_013299825.1 | ASM1329982v1 | MSK.5.17 | Contig | 6/8/2020 | JAAIMS01 |  |
| GCA_013300015.1 | ASM1330001v1 | MSK.19.38 | Contig | 6/8/2020 | JAAING01 |  |
| GCA_013302885.1 | ASM1330288v1 | MSK.7.31 | Contig | 6/8/2020 | JAAIQW01 |  |
| GCA_013303245.1 | ASM1330324v1 | MSK.23.82 | Contig | 6/8/2020 | JAAIRA01 |  |
| GCA_013303495.1 | ASM1330349v1 | MSK.23.46 | Contig | 6/8/2020 | JAAIRJ01 |  |
| GCA_013303515.1 | ASM1330351v1 | MSK.22.53 | Contig | 6/8/2020 | JAAIRM01 |  |
| GCA_013303565.1 | ASM1330356v1 | MSK.17.82 | Contig | 6/8/2020 | JAAIRP01 |  |
| GCA_013303625.1 | ASM1330362v1 | MSK.15.77 | Contig | 6/8/2020 | JAAIRR01 |  |
| GCA_013303695.1 | ASM1330369v1 | MSK.15.32 | Contig | 6/8/2020 | JAAIRV01 |  |
| GCA_013303805.1 | ASM1330380v1 | MSK.11.9 | Contig | 6/8/2020 | JAAIRY01 |  |
| GCA_015553185.1 | ASM1555318v1 | D40t1_170626_D9 | Scaffold | 11/17/2020 | JADNGF01 | 93 |
| GCA_015554725.1 | ASM1555472v1 | 1001285H_161024_A10 | Scaffold | 11/17/2020 | JADNIZ01 | 69 |
| GCA_015557345.1 | ASM1555734v1 | 1001302B_160321_G6 | Scaffold | 11/17/2020 | JADNOC01 | 89 |
| GCA_015559285.1 | ASM1555928v1 | D52t1_170925_A9 | Scaffold | 11/17/2020 | JADMPO01 | 71 |
| GCA_015559445.1 | ASM1555944v1 | BSD2780120874_150323_G6 | Scaffold | 11/17/2020 | JADMPV01 | 53 |
| GCA_015561245.1 | ASM1556124v1 | D31t1_170403_H6 | Scaffold | 11/17/2020 | JADMTD01 | 126 |
| GCA_015561395.1 | ASM1556139v1 | D33t1_170424_E3 | Scaffold | 11/17/2020 | JADMTQ01 | 278 |
| GCA_015666995.1 | ASM1566699v1 | BSD2780061687_150420_H1 | Scaffold | 11/23/2020 | JADPDH01 | 68 |
| GCA_016904015.1 | ASM1690401v1 | FDAARGOS_1342 | Complete Genome | 2/17/2021 |  | 1 |
| GCA_030167985.1 | ASM3016798v1 | CC55_001C | Complete Genome | 6/5/2023 |  | 1 |
| GCA_902167785.1 | Ruminococcus_gnavus_TS_8243C_mod2 |  | Contig | 7/15/2019 | CABHNE01 |  |
| GCA_902374425.1 | MGYG-HGUT-01380 |  | Scaffold | 8/16/2019 | CABKQB01 | 41 |
| GCA_015554725.1 | ASM1555472v1 | 1001285H_161024_A10 | Scaffold | Nov, 2020 | JADNIZ01 | 69 |
| GCA_001406655.1 | GCF_001406655.1 | 2789STDY5608852 (H2_28) | Scaffold | Oct, 2015 | CYZG01 | 50 |
| GCA_003603715.1 | ASM360371v1 | TM07-2AC | Scaffold | 10/1/2018 | QVHG01 | 69 |
| GCA_001304955.1 | ASM130495v1 | UC5_1-2D9 | Contig | 9/25/2015 | BBZS01 |  |
| Sequenced in this study | | 32-6-I_9_D6_FAA |  |  |  |  |
| Sequenced in this study | | 6NTP-36TB |  |  |  |  |
| Sequenced in this study | | UC5_1-2D9 |  |  |  |  |
| Sequenced in this study | | MCC5204 |  |  |  |  |
| Sequenced in this study | | MCC5206 |  |  |  |  |
| Sequenced in this study | | MCC4812 |  |  |  |  |
| Sequenced in this study | | MCC5216 |  |  |  |  |
| Sequenced in this study | | MCC5217 |  |  |  |  |
| Sequenced in this study | | MCC3901 |  |  |  |  |
| Sequenced in this study | | MCC5207 |  |  |  |  |
| Sequenced in this study | | MCC5215 |  |  |  |  |
| Sequenced in this study | | MCC5211 |  |  |  |  |
| Sequenced in this study | | MCC5209 |  |  |  |  |
| Sequenced in this study | | MCC3209 |  |  |  |  |
| Sequenced in this study | | MCC3904 |  |  |  |  |
| Sequenced in this study | | MCC3903 |  |  |  |  |
| Sequenced in this study | | MCC5202 |  |  |  |  |
| Sequenced in this study | | MCC4811 |  |  |  |  |
| Sequenced in this study | | MCC3900 |  |  |  |  |
| Sequenced in this study | | MCC5210 |  |  |  |  |

Supplementary Table 3. Assembly metrics for *R. gnavus* isolates sequenced in this study.

| Strain | Genome size (bp) | % GC | Contigs | N50 (bp) | Mean contig length (bp) | Longest contig (bp) |
| --- | --- | --- | --- | --- | --- | --- |
| MCC3900 | 3059838 | 42.99083807704852 | 107 | 132083 | 28596 | 301847 |
| MCC 3901 | 3085472 | 42.907146783377065 | 82 | 220109 | 37627 | 647129 |
| MCC3902 | 3520144 | 43.038921135044475 | 261 | 52850 | 13487 | 262942 |
| MCC3903 | 3155143 | 42.507962396633054 | 116 | 122436 | 27199 | 591635 |
| MCC3904 | 3115609 | 42.89726342426152 | 101 | 126011 | 30847 | 635123 |
| MCC4811 | 3057466 | 42.99066612678604 | 112 | 132083 | 27298 | 300335 |
| MCC4812 | 3704944 | 42.17580616603112 | 175 | 124232 | 21171 | 397372 |
| MCC5202 | 3140521 | 42.52737045859588 | 140 | 188701 | 22432 | 849948 |
| MCC5204 | 3299472 | 42.930202165679844 | 147 | 135285 | 22445 | 272348 |
| MCC5206 | 3541765 | 42.497398895748304 | 162 | 128307 | 21862 | 393806 |
| MCC5207 | 3082785 | 42.92002199310039 | 159 | 139569 | 19388 | 308993 |
| MCC5209 | 3338680 | 42.76684198545533 | 136 | 106236 | 24549 | 219546 |
| MCC5210 | 2999057 | 43.06463665078723 | 118 | 137248 | 25415 | 281615 |
| MCC5211 | 3599071 | 42.14584819 | 160 | 132457 | 22494 | 272403 |
| MCC5215 | 3156708 | 42.40053245342933 | 70 | 200347 | 45095 | 472844 |
| MCC5216 | 3371647 | 42.75109464306317 | 167 | 155748 | 20189 | 277486 |
| MCC5217 | 3253255 | 42.620052839387014 | 168 | 97635 | 19364 | 200067 |
| 6NTP-36TB | 3460228 | 42.66369152552953 | 117 | 289780 | 29574 | 747057 |
| 32-6-I_9_D6_FAA | 3471424 | 42.49961975258568 | 297 | 80681 | 11688 | 255243 |

Supplementary Table 4. Summary of *R. gnavus* genome quality as assessed using CheckM^6^. All strains are marker lineage f__Lachnospiraceae (UID1256) for the taxonomic rank of the lineage-specific marker set used to estimated genome completeness, contamination, and strain heterogeneity.

| Strain | (a)  # genomes | (b)  # markers | (c)  # marker sets | (d)  0 | (d)1 | (d)2 | (d)  3 | (d)4 | (d)5+ | (e) Completeness | (f)  Contamination | (g)  Strain heterogeneity |
| --- | --- | --- | --- | --- | --- | --- | --- | --- | --- | --- | --- | --- |
| MCC3900 | 33 | 333 | 171 | 1 | 332 | 0 | 0 | 0 | 0 | 99.42 | 0 | 0 |
| MCC3901 | 33 | 333 | 171 | 1 | 332 | 0 | 0 | 0 | 0 | 99.42 | 0 | 0 |
| MCC3902 | 33 | 333 | 171 | 1 | 330 | 2 | 0 | 0 | 0 | 99.42 | 0.88 | 0 |
| MCC3903 | 33 | 333 | 171 | 1 | 330 | 2 | 0 | 0 | 0 | 99.42 | 0.39 | 0 |
| MCC3904 | 33 | 333 | 171 | 1 | 332 | 0 | 0 | 0 | 0 | 99.42 | 0 | 0 |
| MCC4811 | 33 | 333 | 171 | 1 | 332 | 0 | 0 | 0 | 0 | 99.42 | 0 | 0 |
| MCC4812 | 33 | 333 | 171 | 1 | 329 | 3 | 0 | 0 | 0 | 99.42 | 0.97 | 0 |
| MCC5202 | 33 | 333 | 171 | 1 | 330 | 2 | 0 | 0 | 0 | 99.42 | 0.68 | 50 |
| MCC5204 | 33 | 333 | 171 | 1 | 332 | 0 | 0 | 0 | 0 | 99.42 | 0 | 0 |
| MCC5206 | 33 | 333 | 171 | 1 | 332 | 0 | 0 | 0 | 0 | 99.42 | 0 | 0 |
| MCC5207 | 33 | 333 | 171 | 1 | 332 | 0 | 0 | 0 | 0 | 99.42 | 0 | 0 |
| MCC5209 | 33 | 333 | 171 | 1 | 332 | 0 | 0 | 0 | 0 | 99.42 | 0 | 0 |
| MCC5210 | 33 | 333 | 171 | 1 | 332 | 0 | 0 | 0 | 0 | 99.42 | 0 | 0 |
| MCC5211 | 33 | 333 | 171 | 1 | 330 | 1 | 1 | 0 | 0 | 99.42 | 1.27 | 0 |
| MCC5215 | 33 | 333 | 171 | 1 | 331 | 1 | 0 | 0 | 0 | 99.42 | 0.19 | 0 |
| MCC5216 | 33 | 333 | 171 | 1 | 332 | 0 | 0 | 0 | 0 | 99.42 | 0 | 0 |
| MCC5217 | 33 | 333 | 171 | 1 | 331 | 1 | 0 | 0 | 0 | 99.42 | 0.58 | 0 |
| 6NTP-36TB | 33 | 333 | 171 | 1 | 332 | 0 | 0 | 0 | 0 | 99.42 | 0 | 0 |
| 32-6-I_9_D6_FAA | 33 | 333 | 171 | 1 | 331 | 1 | 0 | 0 | 0 | 99.42 | 0.1 | 0 |

(a) number of reference genomes used to infer the lineage-specific marker set

(b) number of marker genes within the inferred lineage-specific marker set

(c) number of co-located marker sets within the inferred lineage-specific marker set

(d) 0-5+: number of times each marker gene is identified

(e) estimated completeness of genome as determined from the presence/absence of marker genes and the expected colocalization of these genes

(f) estimated contamination of genome as determined by the presence of multi-copy marker genes and the expected colocalization of these genes

(g) estimated strain heterogeneity as determined from the number of multi-copy marker pairs which exceed a specified amino acid identity threshold

References:

1. Rogers LA. THE AMERICAN TYPE-CULTURE COLLECTION. Science 1925; 62:267.

2. Baker R, Peacock S. BEI Resources: supporting antiviral research. Antiviral Res 2008; 80:102–6.

3. Sandborn WJ, Panés J, Danese S, Sharafali Z, Hassanali A, Jacob-Moffatt R, Eden C, Daperno M, Valentine JF, Laharie D, et al. Etrolizumab as induction and maintenance therapy in patients with moderately to severely active Crohn’s disease (BERGAMOT): a randomised, placebo-controlled, double-blind, phase 3 trial. The Lancet Gastroenterology & Hepatology 2023; 8:43–55.

4. Sandborn WJ, Vermeire S, Tyrrell H, Hassanali A, Lacey S, Tole S, Tatro AR. Etrolizumab for the treatment of Ulcerative Colitis and Crohn’s disease: An overview of the phase 3 clinical program. Adv Ther 2020; 37:3417–31.

5. Hall AB, Yassour M, Sauk J, Garner A, Jiang X, Arthur T, Lagoudas GK, Vatanen T, Fornelos N, Wilson R, et al. A novel Ruminococcus gnavus clade enriched in Inflammatory Bowel Disease patients. Genome Med 2017; 9:103.

6. Parks DH, Imelfort M, Skennerton CT, Hugenholtz P, Tyson GW. CheckM: assessing the quality of microbial genomes recovered from isolates, single cells, and metagenomes. Genome Res. 2015;25(7):1043-1055.

**Supplementary Materials and Methods**

*gBlock sequence for assembling NanH expression vector*

The following gBlock encoding *R. gnavus* NanH, codon-optimized for *E. coli*, was cloned into pET11a pre-digested with NdeI/BamHI, by Gibson assembly:

GTTTAACTTTAAGAAGGAGATATACATATGGCACACCATCATCACCATCATAGTAGCGGCCTGGAAAACTTATACT



**NdeI**

**6x His**

**TEV cleavage site**

TCCAAGGCTCGAGCCAAGAAGCGCAAACAGACGTGATAGAAGCAGTTGCAGAGAAAAAGCAAGATACAGAGAG



**5’ *nanH* coding sequence (no signal sequence, residues 1-25)**

TTCCTCTGTTCCTGTCTTGCAGAAAGAAGGGATTGAAATCTCAGAGGGTACCGGATACGATTTGTCAAAAGAGCCGGGGGCCGCCACTGTTAAGGCATTAGAACAGGGCACCATCGTGATCTCATATAAGACAACGTCCGAGAATGCCATACAATCACTTCTTTCGGTGGGTAATGGTACGAAGGGGAACCAGGATAGACACTTTCATCTTTACATAACAAATGCTGGCGGAGTCGGTATGGAATTGAGAAACACCGATGGCGAGTTTAAGTATACCCTGGACTGCCCTGCTGCGGTTCGTGGATCTTATAAGGGGGAACGTGTCTCCAATACGGTGGCACTTAAGGCGGACAAGGAGAATAAACAGTACAAATTATTCGCCAACGGTGAACTTATAGCGACGTTGGACCAAGAAGCATTCAAATTCATAAGCGATATAACCGGAGTAGATAACGTAATGCTGGGAGGCACTATGCGGCAAGGAACAGTGGCCTACCCCTTCGGTGGGTCCATAGAGCGGATGCAAGTCTATCGGGATGTGTTGTCGGACGACGAGCTTATTGCCGTTACAGGCAAAACAATTTATGCTGAAAATATCTTTTACGCTGGGGATGCCACGAAAAGTAATTATTTTAGAATTCCGAGCTTGTTAGCGCTTGACTCCGGCACCGTGATCGCGGCCGCGGACGCACGCTACGGTGGGACACACGATGCGAAGTCGAAAATCAATACGGCCTTTGCAAAAAGCACAGATGGAGGGAAAACATGGGGGCAGCCCACTTTACCATTGAAATTCGATGACTATGTAGCAAAGAACATTGACTGGCCGCGCGATAGCGTAGGAAAAAACGTGCAAATCCAGGGAAGTGCATCTTACATAGACCCTGTACTTCTGGAAGATAAGGAGACACACCGGGTATTCCTGTTCGCAGATATGATGCCCGCAGGCATTGGAAGTTCAAATGCTTCGGTCGGGTCTGGGTTTAAAGAGGTCGATGGTAAGAAATACCTTAAGCTTCACTGGAAGGACGATGCTGCGGGTACGTACGATTACAGCGTGCGGGAAAACGGGACTATTTATAACGATACGACAAACTCCGCGACTGAGTATTCCGTGGACGGGGAATATAATCTTTACAAAAATGGTAATGCAATGTTATGCAAACAATATGATTACAACTTTGAGGGAACCAAATTGCTGGAGACTCAAACTGATACAGATGTCAATATGAACGTGTTCTACAAAGACGCAGACTTTAAAGTATTTCCAACTACCTACTTGGCCATGAAGTATTCCGACGACGAAGGAGAAACGTGGAGTGATCTGCAAATAGTCTCGACCTTCAAGCCGGAGGAAAGCAAGTTTCTTGTATTAGGACCTGGAGTAGGGAAACAAATAGCGAATGGAGAGCATGCCGGTAGACTGATAGTGCCCTTGTATTCGAAATCAAGTGCCGAGTTGGGTTTCATGTACTCGGATGATCACGGTAATAATTGGACGTATGTAGAAGCAGACCAGAATACCGGCGGCGCCACCGCCGAAGCGCAAATCGTGGAAATGCCCGATGGATCGTTGAAAACCTACTTGCGTACAGGGAGTGGGTACATAGCTCAGGTAATGAGTACTGACGGTGGTGAGACATGGTCCGAACGGGTTCCTCTGACGGAGATCGCCACAACAGGTTATGGCACACAGCTTTCAGTCATCAACTACTCTCAGCCGGTGGATGGAAAACCAGCAATATTACTGAGTGCGCCTAATGCGACGAACGGACGGAAGAATGGGAAAATTTGGATTGGATTAATAAGCGAAACTGGCAACTCTGGAAAGGATAAATACTCTGTGGACTGGAAGTACTGCTATTCCGTCGACACGCCGCAGATGGGTTATAGCTATTCTTGTCTTACCGAATTGCCGGATGGAGAAATAGGCCTTCTTTATGAGAAATATGATAGTTGGTCACGGAACGAATTACATTTAAAAAACATTCTTAAGTATGAGCGCTTTAATATTGATGAGCTTAAAGTACAACCTTAAGGATCCGGCTGCTAACAA



***nanH - 3’ end***

**BamHI**

*Chemical analysis of 2,7-anhydro-Neu5Ac synthesis product*

Liquid chromatography-mass spectrometry (LC-MS) analysis was performed using an Agilent 1290 ultra-high-performance liquid chromatography system coupled to an Agilent MSD (6140) mass spectrometer, using electrospray ionization. LC separation was performed using a Phenomenex XB-C18 column (1.7μm, 50 × 2.1 mm), at 40 °C and a flow rate of 0.4 mL/min. Mobile phase A (MPA) was water with 0.1% FA and mobile phase B (MPB) was acetonitrile with 0.1% FA. The LC method consisted of an initial equilibration at 2% MPB for 1.5 min, followed by a gradient from 2 - 98% MPB over 7 min, and terminating in a hold at 98% MPB for 1.5 min. UV absorbance was collected at 220 nm and 254 nm and a full mass spectrometry scan was applied to all experiments. 2,7-anhydro-Neu5Ac m/z = 292.1 [M+H]^+^.

NMR spectra were recorded on a Bruker Avance 500 MHz spectrometer with an HCN cryoprobe. Data were acquired at 298 K in DMSO-d6, with chemical shifts referenced to 2.50 ppm in ^1^H and 39.51 ppm in ^13^C; or at 298 K in D_2_O, with chemical shifts referenced to 4.79 ppm in ^1^H. Chemical shifts are expressed in δ ppm. ^1^H NMR data: (500 MHz, D_2_O) δ 4.55 (s, ^1^H), 4.45 (d, J = 7.8 Hz, ^1^H), 3.96 (d, J = 5.5 Hz, ^1^H), 3.93 (s, ^1^H), 3.77 (dd, J = 11.9, 3.1 Hz, ^1^H), 3.61 (dd, J = 12.0, 6.1 Hz, ^1^H), 3.55 (td, J = 6.8, 2.9 Hz, ^1^H), 2.18 (dd, J = 15.3, 5.6 Hz, ^1^H), 2.05 (s, ^3^H), 2.02 (d, J = 15.7 Hz, ^1^H).

*LC-MS analysis of 2,7-anhydro-Neu5Ac stability in culture medium*

Media samples were quenched with 8:2 methanol:water, sonicated, and centrifuged for protein crush and precipitation. Supernatants were dried down by N_2_ gas and reconstituted in 8:2 acetonitrile:water. LC-MS analysis was carried out using a Shimazu LC system coupled to a Thermo QEplus Obitrap Mass Spectrometer. For LC separation, a Waters BEH Amide column (2.1 mm x 100 mm, 1.7 µm) was used with mobile phase A of water and mobile phase B of 95:5 ACN:water, both buffered with 10 mM ammonium formate and 0.125% formic acid. LC was performed at a flow rate of 0.4 mL/min and 40 ˚C with following gradient: 0 min 100% B, 0.5 min 100% B, 2 min 70 B%, 7.7 min 40% B, 9.5 min 30% B, 10.25 min 30% B, 12.75 min 100 % B, 16.75 min 100% B. MS was operated in negative ion ddMS2 mode to acquire accurate mass, retention time, and MS/MS at a mass accuracy of 5 ppm and mass resolution of 70000.
